# Supplementary material for: Asymmetrizing an icosahedral virus capsid by hierarchical assembly of subunits with designed asymmetry
Source: Nat Commun. 2021 Jan 26;12:589. doi: 10.1038/s41467-020-20862-1 (PMC7838286; doi:10.1038/s41467-020-20862-1)
Supplement: Supplementary file 1 — Supplementary Information [file 41467_2020_20862_MOESM1_ESM.docx]

**Supplementary Information:**

**Asymmetrizing an icosahedral virus capsid by hierarchical assembly of subunits with designed asymmetry**

Zhongchao Zhao^1^, Joseph Che-Yen Wang^1,2,3^, Mi Zhang^4^, Nicholas A. Lyktey^4^, Martin F. Jarrold^4^, Stephen C. Jacobson^4^, and Adam Zlotnick^1^*

1 Molecular and Cellular Biochemistry Department, Indiana University, Bloomington, IN 47405, United States

2 Indiana University Electron Microscopy Center, Indiana University, Bloomington, IN 47405, United States

3 Department of Microbiology & Immunology, Pennsylvania State University College of Medicine, Hershey, PA 17033 United States

4 Department of Chemistry, Indiana University, Bloomington, IN 47405, United States

**Contents:**

**Supplementary Figures
 Supplementary Fig. 1.** Design of a bicistronic plasmid for heterodimer expression. **Supplementary Fig. 2.** Assembly of Cp149 homodimer and Cp149_His_Cp149_Y132A_ heterodimer.

**Supplementary Fig. 3.** Negative stain micrograph showing hexamers and double hexamers.
 **Supplementary Fig. 4.** Cp150 can co-assemble with Cp149_His_Cp149_Y132A_ dimers.
 **Supplementary Fig. 5.** A negative stain micrograph of Cp149HisCp149Y132A heterodimers and Cp150 dimers co-assembled capsids.
 **Supplementary Fig. 6.** Disassembly of hexamer-nucleated capsids into holey capsids.

**Supplementary Fig. 7.** Apparatus and data for resistive-pulse sensing of hexamer-nucleated, holey, and refilled capsids.

**Supplementary Fig. 8.** Current traces from multicycle resistive-pulse sensing (i.e., particle ping-pong).

**Supplementary Fig. 9.**  A typical RPS histogram of a mixture of purified T=3 and purified T=4 particles.

**Supplementary Fig. 10.** Chromatographs of absorbance 280 nm (a) and 504 nm (b) show BODIPY co-eluting with refilled capsids and Cp150Bo capsids.

**Supplementary Table 1.** Statistics for negative stain EM data collection and processing

**Supplementary Table 2.** Statistics for cryo-EM data collection and processing


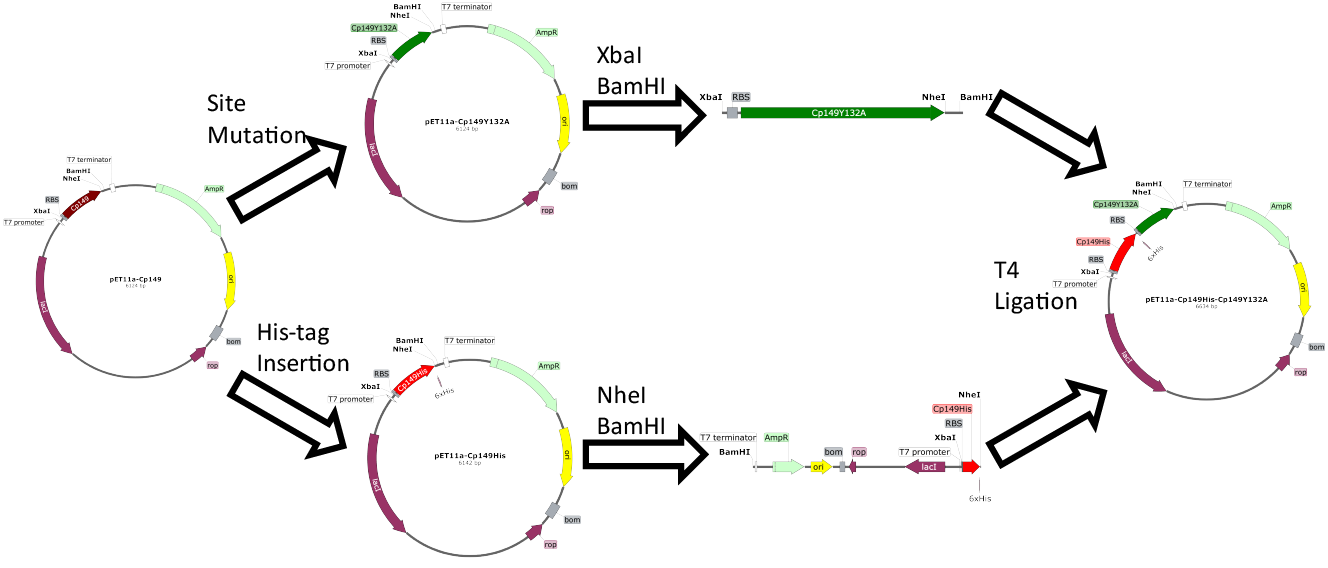


**Supplementary Fig. 1.** Design of a bicistronic plasmid for heterodimer expression. Using the expression plasmid pET11a-Cp149, two new plasmids, pET11a-Cp149_His_ and pET11a-Cp149_Y132A_ are generated. By cloning the two HBV core protein monomer genes in tandem after one T7 promoter, a bicistronic plasmid pET11a-Cp149_His_-Cp149_Y132A_ is created.


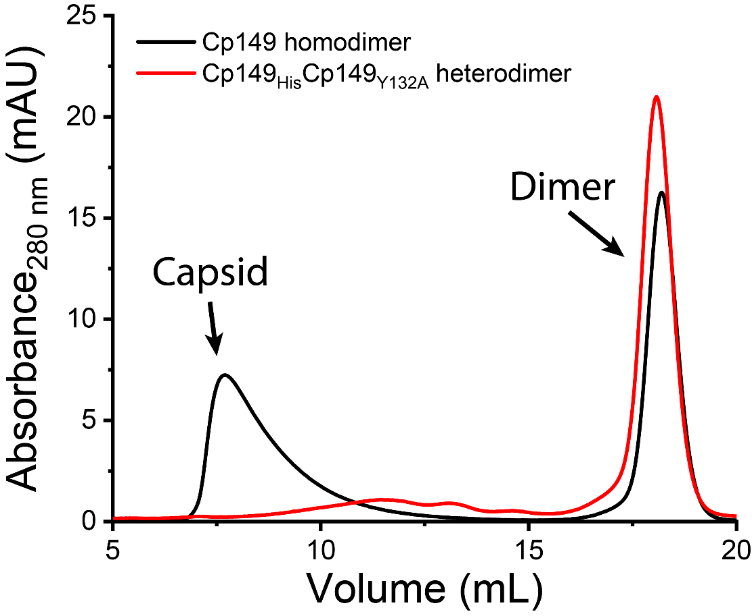


**Supplementary Fig. 2.** Assembly of Cp149 homodimer and Cp149_His_Cp149_Y132A_ heterodimer. Due to the assembly incompetent mutation, Y132A, heterodimer (red) cannot assemble into capsids under the same high ionic strength conditions (300 mM NaCl) as wild-type Cp149 dimers (black).


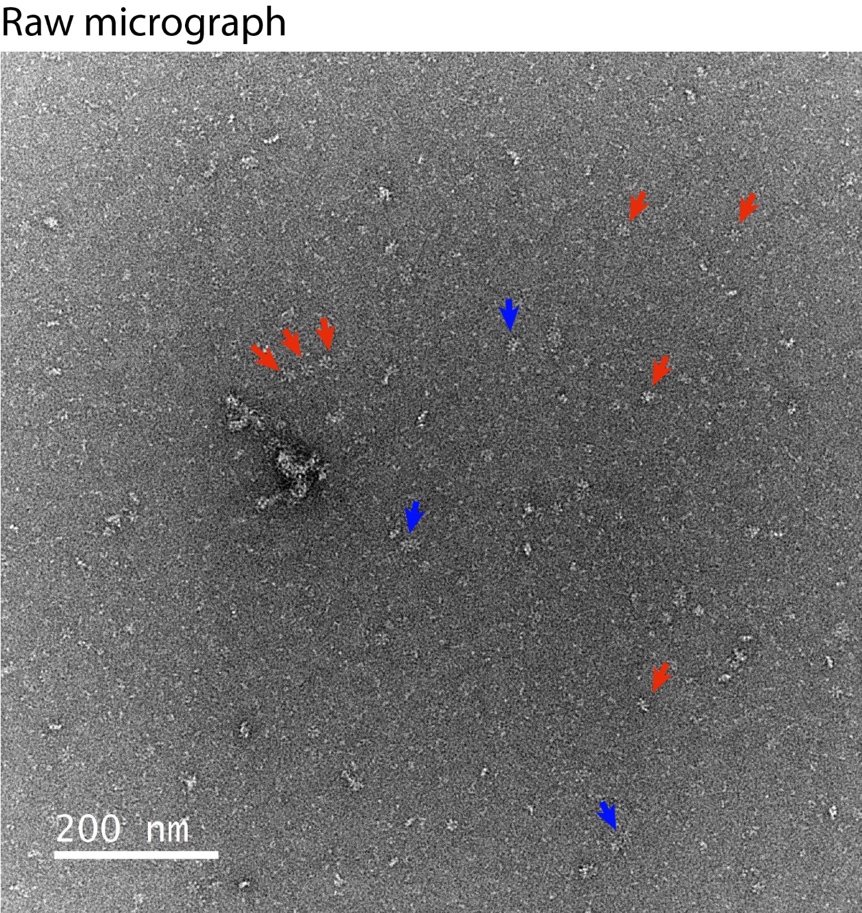


**Supplementary Fig. 3.**  Negative stain micrograph showing hexamers (red arrows) and double hexamers (blue arrows).


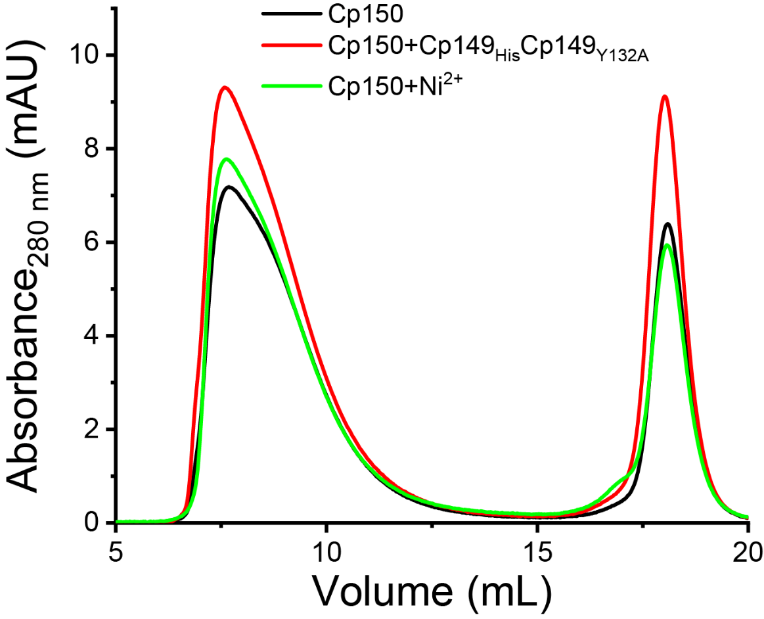


**Supplementary Fig. 4.** Cp150 can co-assemble with Cp149_His_Cp149_Y132A_ dimers. Compared to Cp150 assembly alone (black), the addition of Cp149_His_Cp149_Y132A_ dimers leads to more capsids (red). Whereas, Ni^2+^ shows no effect on Cp150 assembly (green).


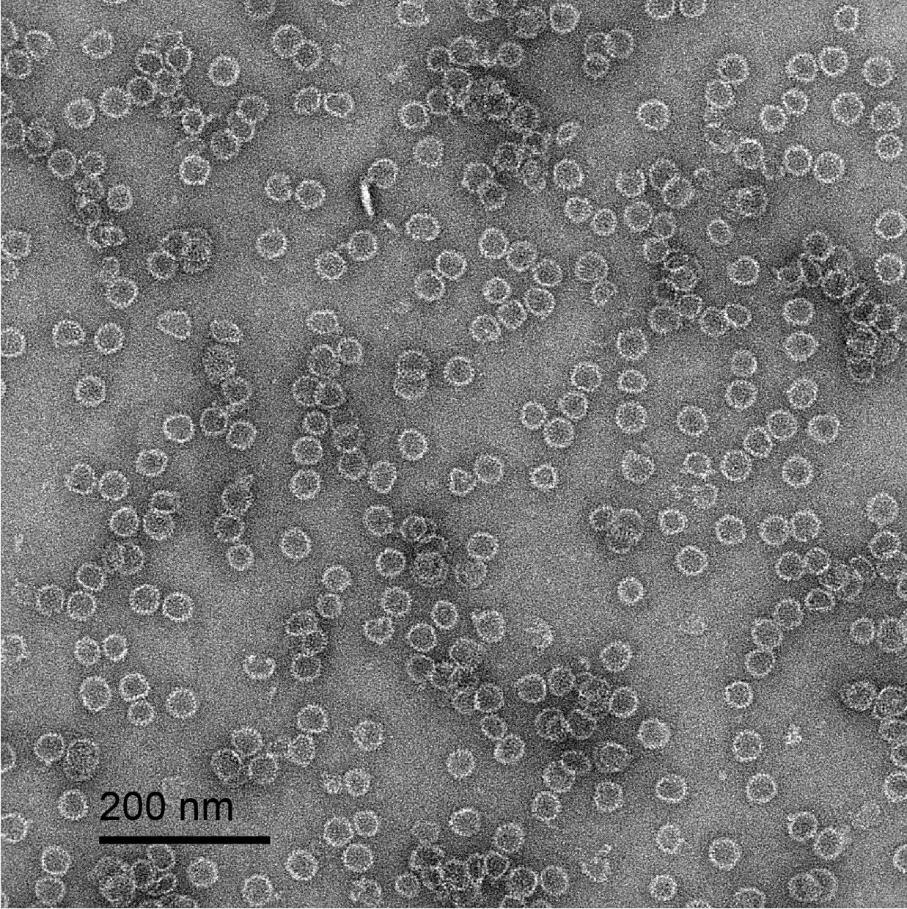


**Supplementary Fig. 5.** A negative stain micrograph of Cp149HisCp149Y132A heterodimers and Cp150 dimers co-assembled capsids.


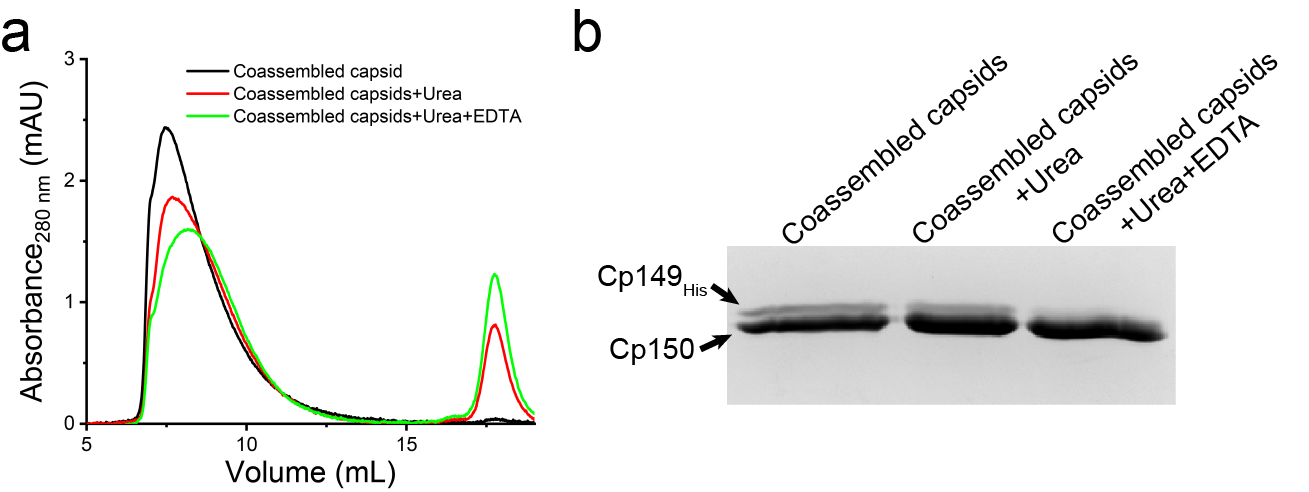


**Supplementary Fig. 6.** Disassembly of hexamer-nucleated capsids into holey capsids. **a,** SEC shows that urea and EDTA treatment of co-assembled capsids leads to release of more dimers (green) than urea treatment alone (red). **b,** Urea and EDTA treatment significantly reduce the Cp149_His_ monomer on holey capsids, indicating the loss of heterodimer hexamers from the co-assembled capsids.


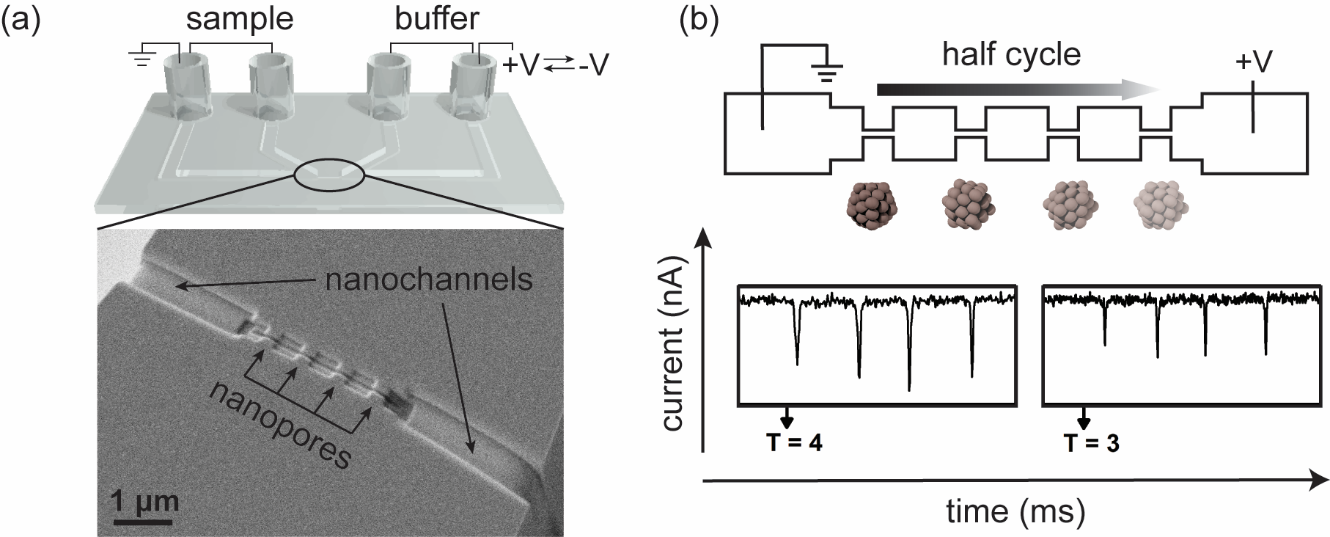


**Supplementary Fig. 7.** Apparatus and data for resistive-pulse sensing of Cp150 standard, hexamer-nucleated, holey, and refilled capsids. (a) Schematic and SEM image of the nanofluidic device used for multicycle resistive-pulse sensing (i.e., particle ping-pong) ^1^. Capsids in the sample reservoirs were electrokinetically driven through the sensing region composed of four nanopores in series. Each of these nanopores was approximately 60 nm deep, 60 nm wide, and 300 nm long. (b) To trap particles during the ping-pong experiments, the potential applied at the buffer reservoir was automatically cycled back and forth from positive to negative after a four-pulse sequence was detected. A single capsid was driven from back and forth until reaching the designated number of cycles (≥ 10.5 cycles) and measured pulses (≥ 84). Current traces for T = 4 and T = 3 capsids are shown for a half-cycle of measurements.





**Supplementary Fig. 8.** Current traces from multicycle resistive-pulse sensing (i.e., particle ping-pong). (a) Current trace of six Cp150 capsids (labeled 1 to 6) sequentially trapped and measured in the four nanopores in series during a ping-pong experiment. (b) Current trace for a single capsid cycled back and forth 10.5 times generating a total of 84 pulses for each particle by automatically switching the electric potential through a LabVIEW program. (c) Current trace of a capsid traveling through the four nanopores for one cycle where four current pulses are generated in each direction.


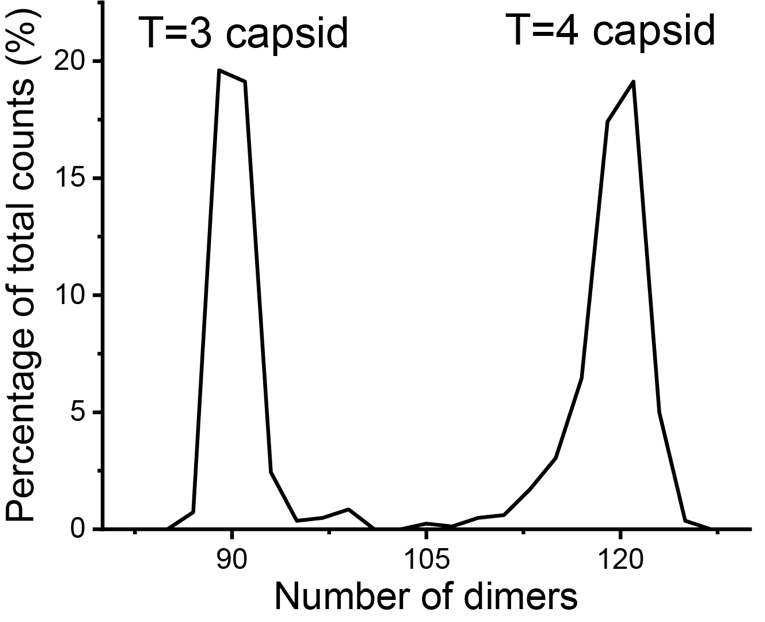


**Supplementary Fig. 9.**  A typical RPS histogram of a mixture of purified T=3 and purified T=4 particles to be used as a standard. Two major species of capsids, T=3 and T=4, are characterized and used to for signal alignment for other tested samples.


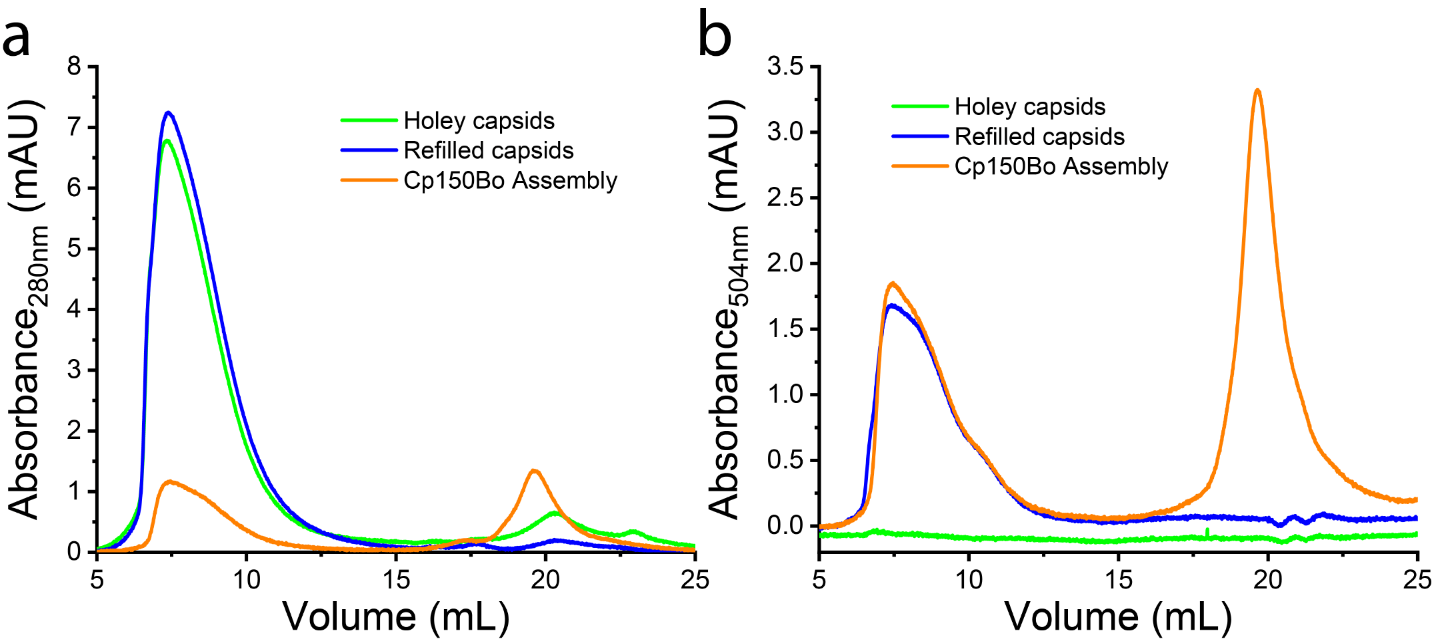


**Supplementary Fig. 10.** Chromatographs of absorbance 280 nm (a) and 504 nm (b) show BODIPY co-eluting with refilled capsids and Cp150Bo capsids. This figure aligns with Fig. 4c.

For the holey capsid sample, we did not observe BODIPY absorbance at 504 nm or fluorescence (Supplementary Fig. 10, and Fig. 4c). For the Cp150Bo assembly control, we observed absorbance at 280 nm and 504 nm for both capsids and dimers. The assembled capsids and the unassembled dimers roughly have the same peak areas in both absorbance chromatographs, indicating they have the same amount of protein. However, we observed little BODIPY fluorescence for capsids compared to the strong fluorescence of unassembled dimers (Fig 4c), which is consistent with the fluorescence quenching that we previously reported for these capsids^2^. However, for the refilled holey capsid sample we observed substantial fluorescence resulting from unquenched fluorophores. As noted in the paper, we did not observe free dimer, indicating they had assembled or were bound into relative high affinity sites.

|  | Cp150Bo at 7.5mL (mAU) | Refilled capsid at 7.5mL (mAU) |
| --- | --- | --- |
| Abs280 | 1.1 | 7.4 |
| Abs504 | 1.9 | 1.7 |
| Abs280/ Abs504 for Cp150Bo | 0.579 | Not relevant |
| A280 attributed to Cp150Bo | 1.1 | 0.98 |

These absorbance data let us quantify the amount of Cp150Bo in the capsid peak. The data were collected on an HPLC equipped with a diode array detector and a fluorescence detector, so that the whole spectrum is measured at one time. Using the A280/A504 for the Cp150Bo capsid peak, we determine an absorbance normalization *n_A280/A50_*. By multiplying the A504 for the refilled capsid peak by *n* we obtain the amount of A280 absorbance related to Cp150Bo. Thus, the fraction of Cp150Bo (*X_Cp150Bo,capsid_*) in the refilled capsid peak is

(1) *X_Cp150Bo,capsid_* = (A504_refilled_ x *n_A280/A50_*) / A280_refilled_

= (1.7 x 0.579) / 7.4

= 0.133

We found that Cp150Bo accounts for ~13% of the absorbance of the refilled capsid peak. If all of these capsids were T=4 particles, this gives an average of ~16 Cp150Bo dimers per capsid. It agrees well with our RPS data and Cryo-EM data, showing a hole missing ~9 to 18 dimers. We cannot exclude the presence of some homogeneous Cp150Bo capsids and some T=3 particles in the refilled holey capsid sample. The loss of the intermediate peak in the RPS of the refilled sample and the loss of free Cp150Bo dimer in the chromatogram, argue that we have found a new repository for Cp150Bo. Therefore, we surmise that the majority of the BODIPY absorbance arose from the refilled Cp150Bo dimers.

**Supplementary Table 1.** Statistics for negative stain EM data collection and processing

| Dataset | Hexamer |
| --- | --- |
| Data collection |  |
| Microscope | JEOL JEM 1400Plus |
| Nominal magnification | 50,000 |
| Voltage (kV) | 120 |
| Electron exposure (e^-^/Å^2^) | 20 |
| Defocus range (µm) | -1.0 to -4.0 |
| Pixel size (Å) | 2.3 (Binned 4.6) |
| Reconstruction (RELION) |  |
| Symmetry imposed | C6 |
| Micrograph number | 165 |
| Initial particle number | 55261 |
| Final particle number | 5755 |
| Map resolution (Å) | 17 |
| FSC threshold | 0.143 |
| EMDB accession code | EMD-22133 [https://www.emdataresource.org/EMD-22133] |

**Supplementary Table 2.** Statistics for cryo-EM data collection and processing

| Dataset | Holey capsid mixture | | |
| --- | --- | --- | --- |
| Data collection |  | | |
| Microscope | Thermo Scientific Talos Artica | | |
| Nominal magnification | 120,000 | | |
| Voltage (kV) | 200 | | |
| Electron exposure (e^-^/Å^2^) | 30 | | |
| Defocus range (µm) | -0.6 to -4.0 | | |
| Pixel size (Å) | 1.128 (Binned 4.512) | | |
| Reconstruction (RELION) |  | | |
| Symmetry imposed | C1 | | |
| Micrograph number | 1366 | | |
| Initial particle number | 65728 | | |
| Capsid type | Complete capsid | Hexamer holey capsid | Double hexamer holey capsid |
| Final particle number | 23478 | 9697 | 7882 |
| Map resolution (Å) | 6.2 | 10.8 | 12.9 |
| FSC threshold | 0.143 | 0.143 | 0.143 |
| EMDB accession code | EMD-22132 | EMD-22134 | EMD-22135 |

EMD-22132 - [https://www.emdataresource.org/EMD-22132]

EMD-22134 - [https://www.emdataresource.org/EMD-22134]

EMD-22135 - [https://www.emdataresource.org/EMD-22135]

**Supplementary References**

1 Zhou, J. *et al.* Characterization of Virus Capsids and Their Assembly Intermediates by Multicycle Resistive-Pulse Sensing with Four Pores in Series. *Anal Chem* **90**, 7267-7274, doi:10.1021/acs.analchem.8b00452 (2018).

2 Stray, S. J., Johnson, J. M., Kopek, B. G. & Zlotnick, A. An in vitro fluorescence screen to identify antivirals that disrupt hepatitis B virus capsid assembly. *Nat Biotechnol* **24**, 358-362 (2006).
